# Molecular Characterization of a Multidrug Resistance IncF Plasmid from the Globally Disseminated *Escherichia coli* ST131 Clone

**DOI:** 10.1371/journal.pone.0122369

**Published:** 2015-04-15

**Authors:** Minh Duy Phan, Brian M. Forde, Kate M. Peters, Sohinee Sarkar, Steven Hancock, Mitchell Stanton-Cook, Nouri L. Ben Zakour, Mathew Upton, Scott A. Beatson, Mark A. Schembri

**Affiliations:** 1 Australian Infectious Diseases Research Centre, School of Chemistry and Molecular Biosciences, The University of Queensland, Brisbane, QLD 4072, Australia; 2 Plymouth University Peninsula Schools of Medicine and Dentistry, Plymouth, United Kingdom; Institut Pasteur, FRANCE

## Abstract

*Escherichia coli* sequence type 131 (*E*. *coli* ST131) is a recently emerged and globally disseminated multidrug resistant clone associated with urinary tract and bloodstream infections. Plasmids represent a major vehicle for the carriage of antibiotic resistance genes in *E*. *coli* ST131. In this study, we determined the complete sequence and performed a comprehensive annotation of pEC958, an IncF plasmid from the *E*. *coli* ST131 reference strain EC958. Plasmid pEC958 is 135.6 kb in size, harbours two replicons (RepFIA and RepFII) and contains 12 antibiotic resistance genes (including the *bla*_CTX-M-15_ gene). We also carried out hyper-saturated transposon mutagenesis and multiplexed transposon directed insertion-site sequencing (TraDIS) to investigate the biology of pEC958. TraDIS data showed that while only the RepFII replicon was required for pEC958 replication, the RepFIA replicon contains genes essential for its partitioning. Thus, our data provides direct evidence that the RepFIA and RepFII replicons in pEC958 cooperate to ensure their stable inheritance. The gene encoding the antitoxin component (*ccdA*) of the post-segregational killing system CcdAB was also protected from mutagenesis, demonstrating this system is active. Sequence comparison with a global collection of ST131 strains suggest that IncF represents the most common type of plasmid in this clone, and underscores the need to understand its evolution and contribution to the spread of antibiotic resistance genes in *E*. *coli* ST131.

## Introduction

*Escherichia coli* sequence type 131 (*E*. *coli* ST131) is a recently emerged and globally disseminated multidrug resistant clone associated with urinary tract and bloodstream infections [1, 2]. *E*. *coli* ST131 was originally identified in 2008 as a major clone linked to the spread of the CTX-M-15 extended-spectrum β-lactamase (ESBL)-resistance gene [3–5]. Since then, *E*. *coli* ST131 has also been strongly associated with fluoroquinolone resistance, as well as co-resistance to aminoglycosides and trimethoprim-sulfamethoxazole [6, 7]. Recent analyses of the global epidemiology of *E*. *coli* ST131 using whole genome sequencing has revealed the CTX-M-15 allele is highly prevalent within a fluoroquinolone resistant-FimH30 (*H*30) ST131 sublineage [8] and demonstrated a significant role for recombination in the evolution of this *E*. *coli* lineage [9].

As observed for most other multidrug resistant *Enterobacteriaciae* pathogens, plasmids are the major vehicles for carriage of antibiotic resistance genes in *E*. *coli* ST131. Multiple plasmids from a range of incompatibility groups and containing various combinations of antibiotic resistance genes, conjugative transfer genes and other cargo genes have been described in *E*. *coli* ST131 strains [2]. This includes the IncF plasmids pEK499, pEK516 [10], pGUE-NDM [11], pC15-1a [12], pJJ1886-5 [13], pEC_B24, pEC_L8, pEC_L46 [14], pJIE186-2 [15], as well as the IncN plasmid pECN580 [16], the IncX plasmid pJIE143 [17] and the IncI plasmid pEK204 [10].

*E*. *coli* EC958 represents one of the best-characterised genome-sequenced *E*. *coli* ST131 strains [18]. *E*. *coli* EC958 is a phylogenetic group B2, CTX-M-15 positive, fluoroquinolone resistant, *H*30 *E*. *coli* ST131 strain [19]. The strain belongs to the pulse field gel electrophoresis defined United Kingdom (UK) epidemic strain A [20], and the recently defined ST131 Clade C2/*H*30-Rx sublineage [8, 9]. *E*. *coli* EC958 contains multiple genes associated with the virulence of extra-intestinal *E*. *coli*, including type 1 fimbriae which are required for adherence to and invasion of human bladder cells, the formation of intracellular bacterial communities, and colonization of the mouse bladder [19, 21]. In animal models, *E*. *coli* EC958 causes acute and chronic urinary tract infection (UTI) [21] and impairment of uterine contractility [22]. *E*. *coli* EC958 is also resistant to the bactericidal action of human serum, and the complement of genes that define this phenotype have been comprehensively defined [23].

*E*. *coli* EC958 contains a large IncF plasmid (pEC958—HG941719) containing multiple antibiotic resistance genes. Here we describe the full annotation of pEC958, and demonstrate that genes encoded on pEC958 are common among other Clade C2/*H*30-Rx ST131 strains. Plasmid pEC958 contains two replicons, and we show that both replicons contribute to its maintenance in *E*. *coli* EC958.

## Materials and Methods

### Bacterial strains and growth conditions

*E*. *coli* EC958 is a UTI strain originally isolated in the UK in 2005 [19]. *E*. *coli* TOP10 has been described previously [24]. *E*. *coli* strains were stored in 15% glycerol at -80°C and routinely cultured at 37°C on solid or in liquid Lysogeny Broth (LB) medium.

### Antimicrobial susceptibility testing

The minimal inhibitory concentrations (MICs) were determined by Etest (bioMérieux Australia) on Mueller-Hinton agar at 37°C. The procedure and interpretation of MIC were performed as recommended by the manufacturer using CLSI breakpoints [25].

### Molecular methods

Plasmid DNA purification was performed using the PureLink HiPure Plasmid Filter Midiprep Kit (Life Technologies). *E*. *coli* TOP10 electro-competent cells were prepared as previously described [23] and pEC958 plasmid DNA was transformed into TOP10 in a 2 mm cuvette using a BioRad GenePulser set to 2.5 kV, 25 mF and 200 Ω. Cells were resuspended in 1 mL SOC medium and incubated at 37°C for 2 hours, then selected on LB agar plates supplemented with ampicillin 100 μg/mL.

### *In silico* replicon sequence typing (RST)

The FAB formula for IncF plasmids (IncF RST scheme [26]) was identified *in silico* using the online pMLST tool (http://cge.cbs.dtu.dk/services/pMLST/) [27]. The pEC958 information was uploaded to the pMLST database (http://pubmlst.org/).

### Annotation of pEC958

The sequence of plasmid pEC958 (emb|HG941719) [18] was manually curated in Artemis [28] using BLAST and literature searches. Antibiotic resistance genes were named in accordance with ResFinder 1.4 [29] and confirmed manually by BLAST and literature searches.

### TraDIS analyses

The TraDIS sequence data used in this work was generated from a previously published study that examined essential genes in EC958 (BioProject number PRJNA189704; input A and B samples) [23]. The short reads were mapped to the pEC958 sequence using Maq version 0.7.1 [30]. Counts of insertion per gene and insertion index were calculated as previously described [23].

### Phylogenetic tree building

The maximum-likelihood phylogenetic tree of EC958_A0140 homologs was built using the PhyML v3.0 online tool [31]. The tree used the WAG model for amino acid substitution and branch supports were calculated using approximate likelihood-ratio test (aLRT) [32].

### Visualization

The read counts and insertion sites from TraDIS were visualized using Artemis version 15.0 [28]. The circular genome diagram was generated by DNAplotter [33] and linear genetic diagrams were constructed using Easyfig version 2.1 [34]. Circos [35] and Circoletto [36] were used to generate the sequence comparison figure. Sequence comparisons of pEC958 against ST131 strains were generated using BLAST Ring Image Generator (BRIG) [37].

## Results

### Characteristics of plasmid pEC958

**Fig 1:**
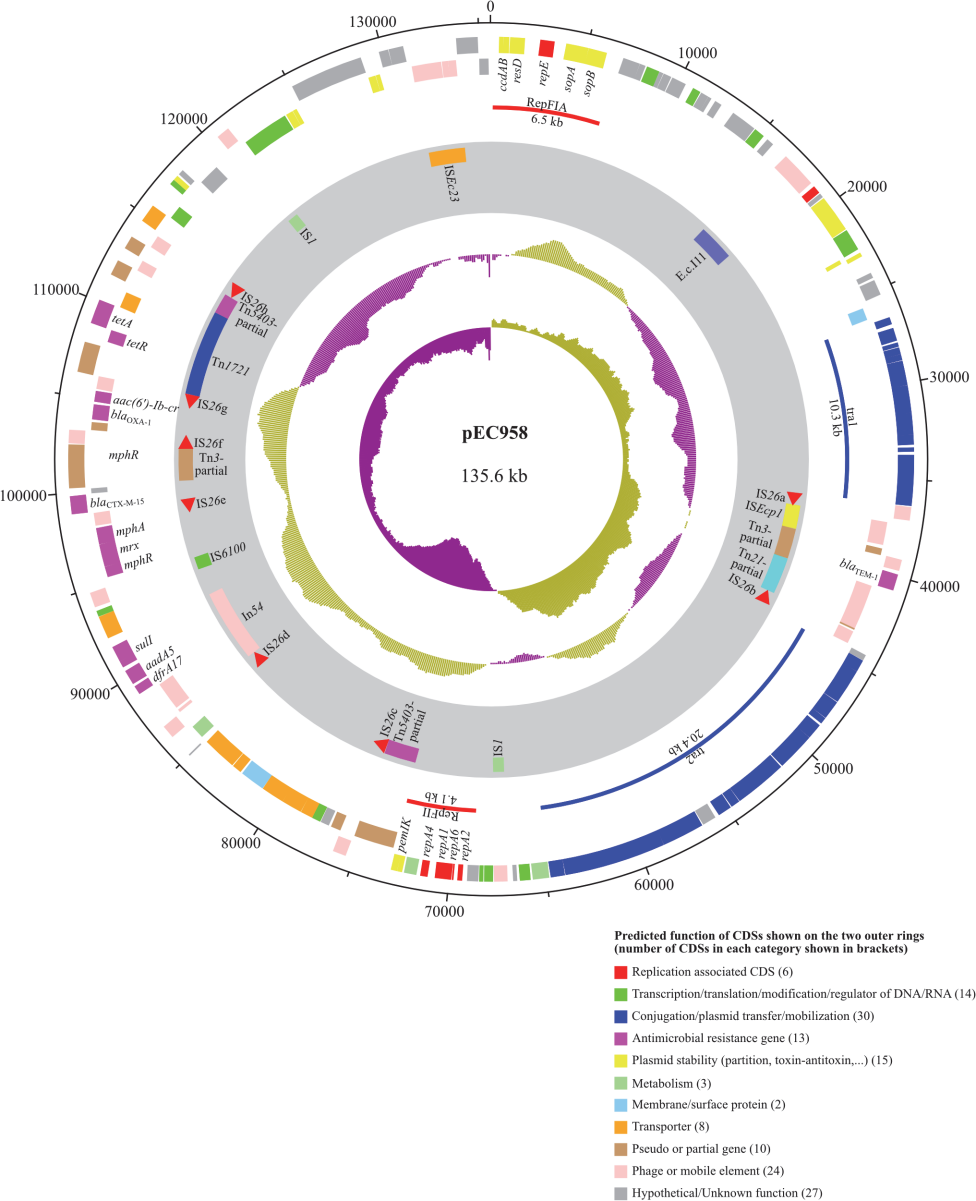
Circular representation of plasmid pEC958. The two outer rings show the coding sequences (CDSs) on the forward and reverse strand of the plasmid. Each CDS is colour-coded by its predicted function as shown in the figure. The grey ring depicts mobile elements identified on the plasmid. The two inner rings represent the GC plot and GC skew graph, respectively.

**Fig 2:**
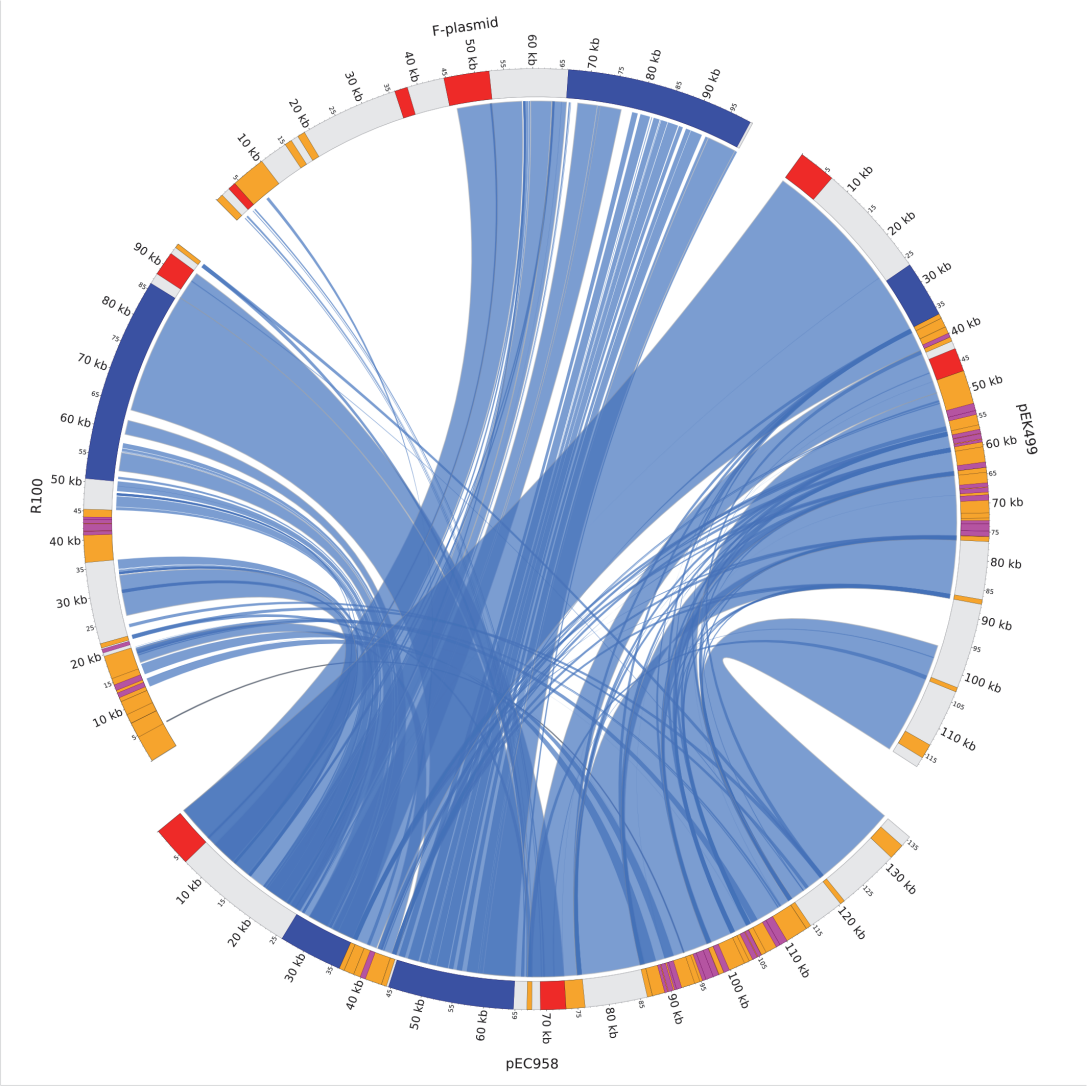
Sequence comparison of pEC958 with other closely related plasmids. Regions on plasmids are colour-coded as followed: red—replicon, blue—conjugation transfer, orange—mobile elements, dark pink—antimicrobial resistance genes.

The plasmid pEC958 is a 135,600 bp circular DNA molecule containing 142 coding sequences (CDSs) and 10 pseudogenes (Fig 1). The most closely related plasmid to pEC958 is pEK499 (99% identity covering 85% of pEC958; pEK499 lacks the second transfer region present in pEC958, which accounts for the remaining 15% of pEC958) (Fig 2). *In silico* replicon sequence typing identified pEC958 as a hybrid plasmid containing both IncFII and IncFIA replicons (FAB formula F2:A1:B-).

### The RepFIA replicon

The 6,509bp RepFIA replicon in pEC958 is 99% identical to the corresponding region on the F-plasmid (nt 45922 to 52516, accession no. NC_002483.1) and 100% identical to two other plasmids isolated from *E*. *coli* ST131 strains, pEK499 (NC_013122.1 [10]) and pJJ1886_5 (NC_022651.1 [13]) (Fig 2). As observed in many other RepFIA sequences, this region does not contain the *repC* gene (replication initiation) found on the F-plasmid. The first region of RepFIA in pEC958 contains two *rfsF* sites (the target sequences of the site-specific resolvase ResD [38]), followed by the *oriV-1* origin of replication, *ccdAB* genes (post-segregational killing), and *resD* (multimer resolution). The second region of RepFIA in pEC958 contains the replication *repE* gene (RepFIA) with its upstream sequences *ssiA* (single strand initiation) and *oriV-2* (including the DnaA boxes, A/T rich region and four iterons), and the downstream *incC* iterons (incompatibility and copy-number control). The third region of RepFIA in pEC958 contains the *sopAB* partition genes and their target centromere-like *sopC* sequence. This is the only partition system found on pEC958. Although this RepFIA replicon contains two origins of replication (*oriV-1* and *oriV-2*), replication is predicted to start unidirectionally from *oriV-2* because the bidirectional replication from *oriV-1* is known to require the missing RepC protein [39, 40].

### The RepFII replicon

The second replicon in pEC958, RepFII (4,068 bp), is 99% identical to the IncFII replicon in the *Shigella flexneri* 2b plasmid R100 (accession no. NC_002134.1, [41]) and 100% identical to the RepFII replicon in the *E*. *coli* ST131 plasmid pEK499 (Fig 1). This replicon encodes the essential gene for its replication, *repA1*, which is regulated by the negative regulator RepA2 (CopB), the non-coding RNA *copA* and the regulatory leading peptide RepA6 [42–44]. The pEC958 RepFII origin of replication (*ori*) is located between *repA1* and *repA4*, consistent with previous descriptions for the initiation of DNA replication from this replicon [42, 45–47]. The *repA4* region is important for plasmid stability and contains the *ter* sites for replication termination [48]. The pEC958 RepII replicon contains the *tir* (transfer inhibition protein [49]) and the type II toxin-antitoxin system *pemI/pemK* [50, 51] downstream of *repA4*.

### The transfer region of pEC958 is not functional

The transfer (*tra*) region of pEC958 is disrupted by a composite mobile element flanked by IS*26*a and IS*26*b, carrying *bla*_TEM-1_ gene (Fig 1). The first half of this *tra* region is 100% identical to the corresponding region on pEK499 (F2:A1:B-), and 99% identical to the corresponding region of several other IncF plasmids including pJJ1886_5 (F2:A1:B-), pEC_L46 (F2:A1:B-), pEC_L8 (F2:A1:B-), pEFC36a (F2:A-:B-) and pChi7122-2 (F11:A-:B-). In contrast, the second half of the pEC958 *tra* region is 100% identical to pC15-1a (F2:A-:B-), R100 (F2:A-:B-), pHN3A11 (F2:A-:B-), pFOS-HK151325 (F2:A-:B-), pXZ (F2:A-:B-), pHK23a (F2:A-:B-), pHK01 (F2:A-:B-) and pEG356 (F2:A-:B-). However, the pEC958 conjugation system is missing three genes, namely *trbI*, *traW* and *traU*. TrbI is an inner membrane protein that affects pilus retraction [52]; TraW is required for F-pilus assembly [52]; and mutations in *traU* significantly reduce plasmid transfer proficiency [53]. Despite repeated attempts, we were unable to demonstrate conjugative transfer of pEC958 to recipient strains, supporting the bioinformatic prediction that its conjugation system is non-functional (data not shown).

### Toxin-antitoxin systems

The pEC958 plasmid encodes four toxin-antitoxin (TA) systems: the *hok/sok* system, the *ccdAB* system encoded within RepFIA, the *pemIK* system encoded within RepFII and the *vagDC* system. The *hok*/*sok* locus encodes a type I TA system including a “host killing” (*hok*) transmembrane protein that damages the cell membrane, a “modulation of killing” (*mok*) and a “suppression of killing” (*sok*) antisense RNA that inhibits translation of *mok* [54]. Both *ccdAB* and *pemIK* belong to type II TA system where the toxin protein is inactivated by direct interaction with the antitoxin protein. The *ccdB* gene encodes for a gyrase inhibitor toxin [55] that kills the cell in the absence of the CcdA anti-toxin, which is unstable and degraded by the Lon protease [56]. PemK is a sequence-specific endoribonuclease that cleaves mRNAs to inhibit protein synthesis [50] whereas PemI blocks the endoribonuclease activity and is also subjected to Lon proteolysis [57].

There are two identical copies of the *vagDC* genes in pEC958. Sequence analysis of VagD revealed a PIN_VapC-FitB (cd09881) domain found in toxins of many bacterial TA systems. VagC contains an antitoxin-MazE (pfam04014) domain. The *vagDC* genes have been shown to be involved in plasmid stability in *Salmonella* Dublin, where VagD inhibits cell division and VagC modulates the activity of VagD [58].

### Mobile genetic elements and antibiotic resistance genes

**Fig 3:**
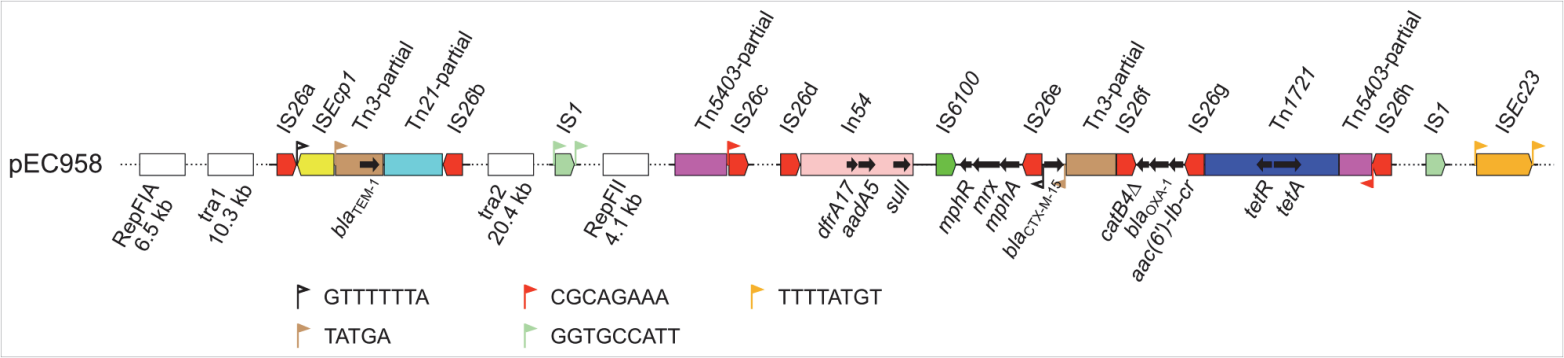
Organizational structure of mobile elements and antibiotic resistance genes on plasmid pEC958. The colour coding is as followed: red—IS*26* (8 copies IS*26*a to IS*26*h), yellow—IS*Ecp1*, brown—Tn*3* (partial), sky blue—Tn*21* (partial), light green—IS*1*, dark pink—Tn*5403*, green—IS*6100*, dark blue—Tn*1721*, orange—IS*Ec23*, dark pink—In*54*. Target duplication sites are indicated by triangle flags. The white blocks represent large regions not detailed in this figure.

The majority of mobile genetic elements and antibiotic resistant genes in pEC958 cluster in two regions: an 8-kb region in the middle of the *tra* region, and a 41-kb region located immediately downstream of the RepFII replicon (Fig 1). Plasmid pEC958 contains eight IS*26* elements (named IS*26*a-IS*26*h), two IS*1* elements, one IS*Ec23* element and one group II intron (E.c.I11, found outside of the two regions) (Fig 3). IS*26*a and IS*26*b are located at the two ends of the 8-kb region, flanking IS*Ecp1*, a remnant of Tn*3*, which includes the *bla*_TEM-1_ gene, and a partial sequence of Tn*21*. The beginning of the 41-kb region contains a partial sequence of Tn*5403* followed by IS*26*c. The region between IS*26*c and IS*26*d contains a cluster of 6 genes (EC958_A0096 to EC958_A0101) predicted to encode a series of ABC transporters and an iron permease. Downstream of IS*26*d is a class I integron In54 [59] with gene cassettes consisting of *dfrA17*, *aadA5* and *sulI*, encoding trimethoprim, streptomycin and sulfonamide resistance, respectively. The *mphR*-*mrx*-*mph(A)* operon encoding resistance to macrolides is located between IS*6100* and IS*26*e. Immediately after IS*26*e is the *bla*_CTX-M-15_ gene encoding cefotaxime resistance. Located between IS*26*f and IS*26*g are *catB4*Δ (non-functional; disrupted by IS*26*f), *bla*_OXA-1_ (beta-lactam resistance) and *aac(6’)-Ib-cr* (fluoroquinolone and aminoglycoside resistance). After IS*26*g lies Tn*1721*, which harbours *tetR* and *tet(A)*, encoding resistance to tetracycline. The end of the 41-kb region contains a partial sequence of Tn*5403* and IS*26*h.

### Functional characterization of antibiotic resistance genes on pEC958

To investigate the antibiotic resistance phenotypes conferred by plasmid pEC958, we transformed the plasmid into *E*. *coli* TOP10. Table 1 shows the resistance profile of wild-type EC958 (which contains pEC958) compared to TOP10(pEC958). EC958 is resistant to 11 of the 18 antibiotics tested, five of which are fully transferable via pEC958. EC958 is resistant to the cephamycin cefoxitin and the three third-generation cephalosporins tested (cefotaxime, ceftazidime and cefpodoxime). These phenotypes, however, were not fully transferred to TOP10 by pEC958. TOP10(pEC958) had elevated MICs to cefoxitin, cefotaxime, ceftazidime and cefpodoxime (MIC of 6, 1.5, 1.5 and 8.0 μg/mL, respectively) compared to the background strain TOP10 (MIC of 4, 0.047, 0.38 and 0.25 μg/mL, respectively), but these MICs were still 6–10 fold lower than those of the EC958 wild-type strain. This suggests that *bla*_CTX-M-15_ on pEC958 plasmid does not mediate the full resistance against third-generation cephalosporins. This is consistent with previous reports of lower resistance to cephalosporins in strains where the *bla*_CTX-M-15_ is separated by IS*26* from its promoter within the IS*Ecp1* element [60–63]. The other resistance phenotypes not transferred were for quinolones and fluoroquinolones. Chromosomal mutations in *gyrA* (S83L, D87N, A828S) and *parC* (S80I, E84V, A192V, A471G, D475E, Q481H) genes are likely to mediate these phenotypes, even though the plasmid carries *aac(6’)-Ib-cr* [64–66].

**Table 1:** Antibiotic resistance profiles (MIC, μg/mL) of EC958 and its pEC958 transformant in *E*. *coli* TOP10.

Antibiotics	Strains		
	EC958	TOP10	TOP10 + pEC958
Ampicillin	≥256 (R)	2 (S)	≥256 (R)
Amoxicillin/Clavulanic Acid	24 (R)	3 (S)	16 (R)
Aztreonam	2 (S)	0.094 (S)	1 (S)
Cefoxitin	48 (R)	4 (S)	6 (S)
Cefotaxime	12 (R)	0.047 (S)	1.5 (I)
Ceftazidime	16 (R)	0.38 (S)	1.5 (S)
Cefpodoxime	48 (R)	0.25 (R)	8.0 (R)
Imipenem	0.25 (S)	0.19 (S)	0.25 (S)
Meropenem	0.125 (S)	0.064 (S)	0.094 (S)
Nalidixic Acid	≥256 (R)	0.38 (S)	0.50 (S)
Ciprofloxacin	≥32 (R)	0.003 (S)	0.008 (S)
Sulfamethoxazole/Trimethoprim	≥32 (R)	0.032 (S)	≥32 (R)
Kanamycin	≥256 (R)	2 (S)	≥256 (R)
Amikacin	24 (I)	3 (S)	24 (I)
Tetracycline	≥256 (R)	0.5 (S)	192 (R)
Tigecycline	0.38 (S)	0.094 (S)	0.38 (S)
Fosfomycin	0.5 (S)	0.38 (S)	0.38 (S)
Nitrofurantoin	4 (S)	0.094 (S)	0.094 (S)

### Genes required for the stable maintenance of pEC958

**Fig 4:**
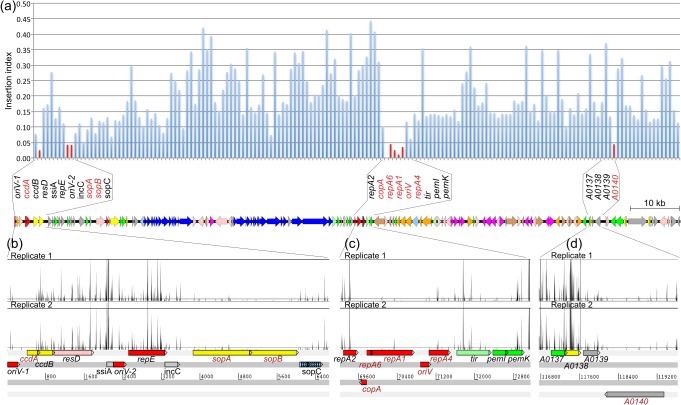
Overview of the TraDIS screen for the identification of pEC958 essential genes. (A) Graph showing the insertion index of each gene on pEC958 (top) in relation to the overall genetic organization of the plasmid (bottom). Nine essential genes (indicated in red) were identified that possessed an insertion index lower than 0.05. (B, C, D) Schematic showing the frequency of Tn insertions mapping to specific regions of pEC958. Essential genes required for the stable maintenance of pEC958 possessed a significantly reduced number of insertions.

In order to gain insights into molecular mechanisms of plasmid stability, we analyzed the TraDIS data from a saturated transposon mutant library of EC958 [23] against the complete sequence of pEC958 to identify genes required for plasmid stability. We used a total of 12 million transposon-tagged reads, of which 901,588 reads (7.4%) were mapped to plasmid pEC958, identifying 27,317 unique insertion sites (i.e. one insertion site every 4.96 bp). To devise a biological threshold for the identification of genes required for the stable maintenance of pEC958, the insertion index (number of mapped reads normalized by gene length) of each plasmid gene was calculated and compared with the *sopAB* genes, which are known to be essential for plasmid partitioning (Fig 4).

A total of 9 genetic elements were identified to be required for the stable maintenance of pEC958. They are the *ccdA*, *sopA* and *sopB* genes in RepFIA; the *copA*, *repA6*, *repA1*, *repA4* genes and the *oriV* region in RepFII; and the hypothetical gene EC958_A0140. Our results indicate that replication of pEC958 is initiated at the *oriV* of RepFII and requires at least the *copA*, *repA6*, *repA1*, *repA4* genes. While RepFIA is not essential for replication, it is required for partitioning (*sopAB*) of pEC958 into daughter cells. Our data also demonstrated that the *ccdAB* TA system located within RepFIA is functional.

**Fig 5:**
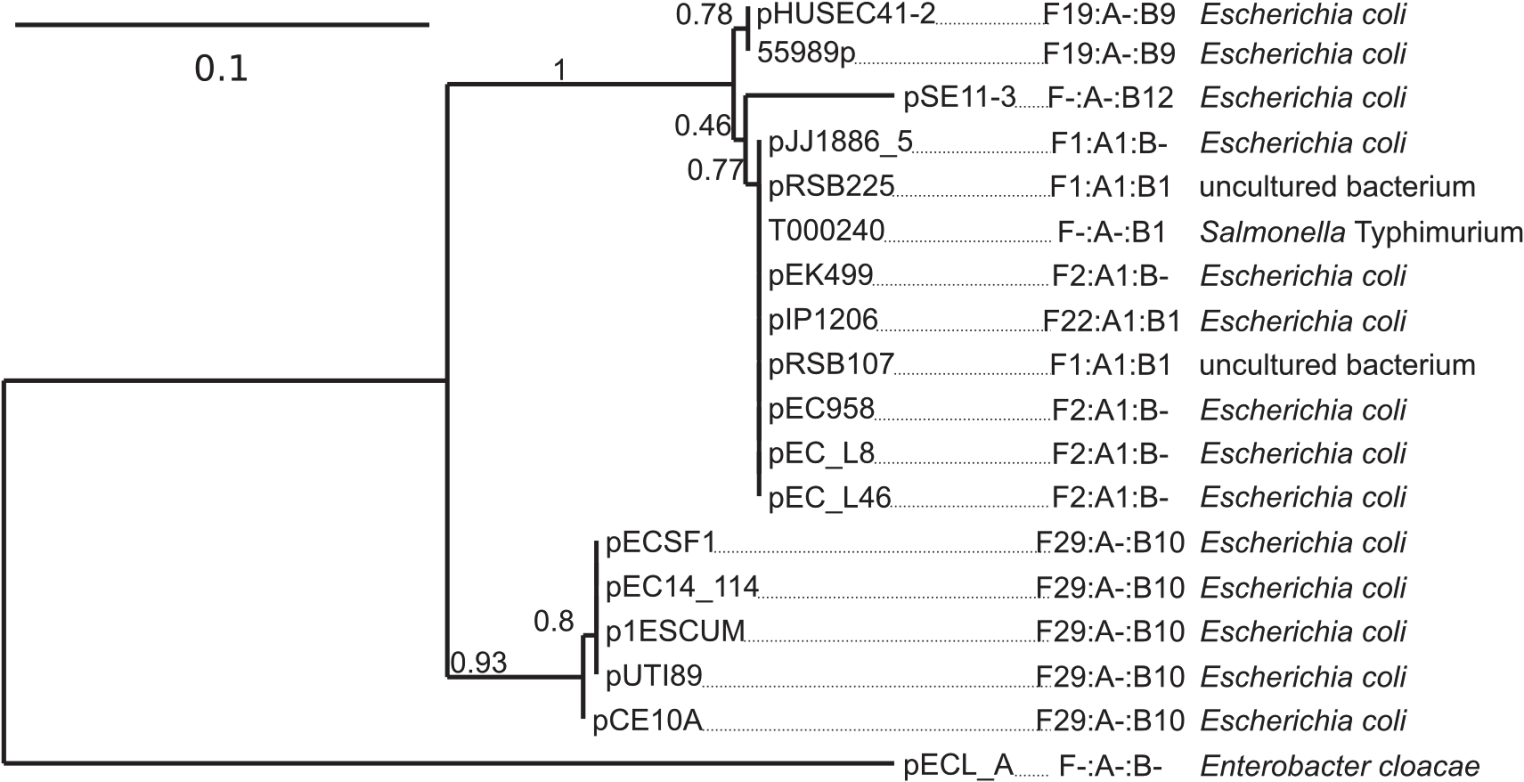
Maximum-likelihood phylogenetic tree showing the relationship of EC958_A0140 translated amino acid sequences. EC958_A0140 sequenced are labeled by plasmid name. Also shown is the replicon nomenclature for each plasmid according to the FAB scheme and the parent organism.

EC958_A0140 represents a novel gene associated with plasmid maintenance. We screened the NCBI complete plasmid sequence database and identified 17 other plasmids that also contain this gene (Fig 5). All of these plasmids were IncF type except for pECL_A (non-typable), and several were also isolated from *E*. *coli* ST131 strains (pJJ1886_5, pEK499, pEC_L8 and pEC_L46). Bioinformatic analysis of EC958_A0140 did not yield any clues regarding is function, and thus further work is required to confirm its role in plasmid stability.

### pEC958-like plasmid sequences are highly prevalent in ST131

**Fig 6:**
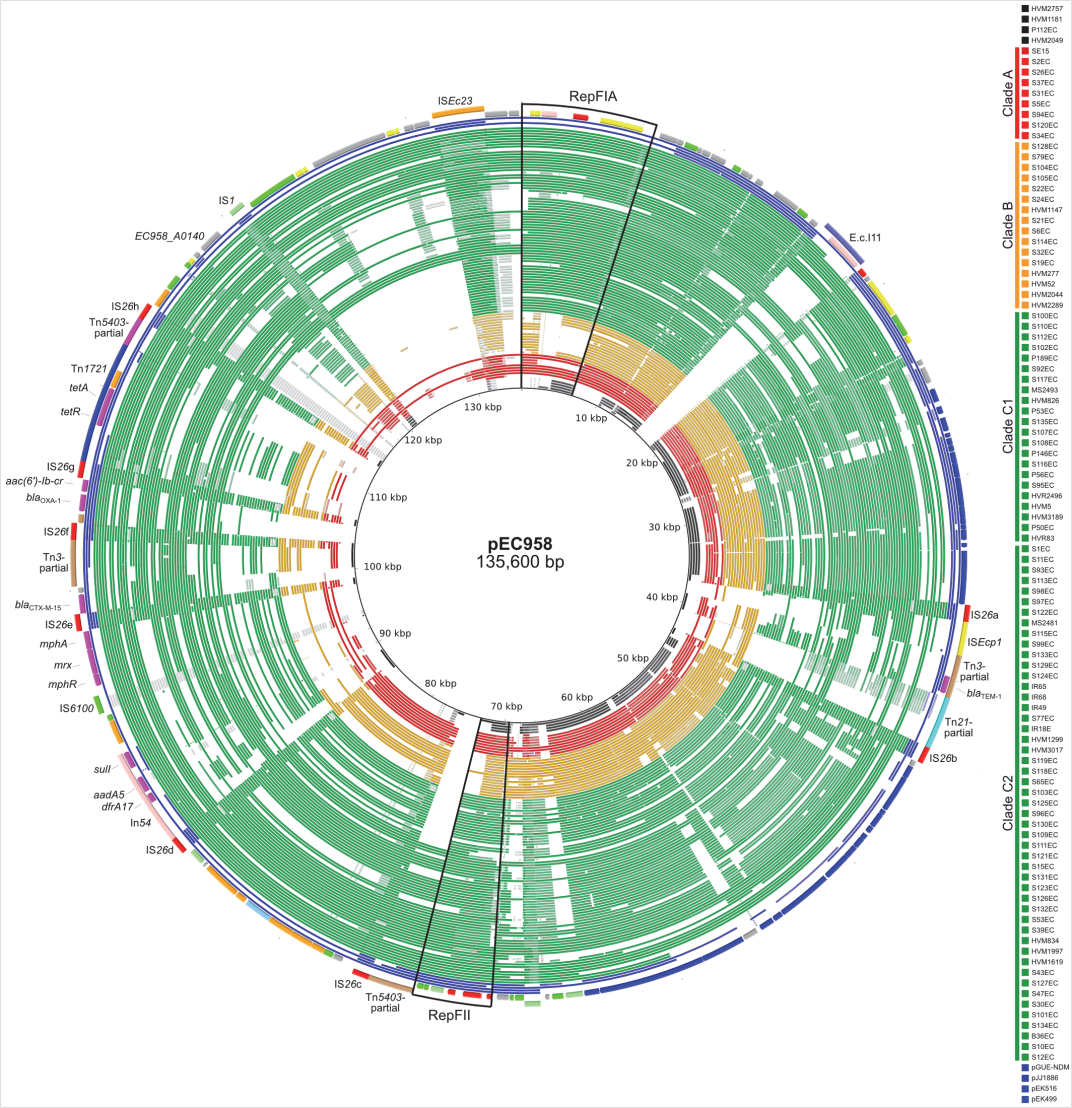
BRIG image depicting the presence of pEC958-like sequences in *E*. *coli* ST131 strains. The strains are coloured according to their previously defined phylogenetic relationship: red (Clade A), orange (Clade B) and green (Clade C) [9]. The degree of coloured shading indicates the level of identity according to BLASTn between pEC958 (nucleotide position highlighted on the inner circle) and the draft Illumina assemblies of the *E*. *coli* ST131 strains [9]. BLASTn matches are coloured based on a nucleotide identity of between 70% and 90% (dark shading = high identity, light shading = low identity). Blank spaces in each ring represent BLASTn matches to pEC958 with less than 70% nucleotide identity, or pEC958 regions with no BLAST matches. Four strains originally characterised as ST131 but later shown to be ST95 are shown in black. Highlighted on the outer ring are the RepFII and RepFIA replicons, as well as antibiotic resistance genes, transposons and IS elements.

The prevalence of pEC958-like plasmid sequences was assessed in a previously described global collection of 97 *E*. *coli* ST131 strains [9]. Fig 6 shows the overview of plasmid sequences from 97 ST131 strains plus four complete ST131 plasmids available on GenBank in comparison with the pEC958 sequence. There are 20 strains and 2 database plasmids (pEK499 and pJJ1886_5) that contain more than 70% of pEC958 gene content, all of which belong to the clade C sublineage C2 (40%) (Fig 6 and S1 Table). Twelve out of these 20 strains (plus pEK499) also harbor all 9 pEC958 essential genes identified above.

*In silico* replicon sequence typing of IncF plasmids was also performed on the 97 strains. Table 2 shows the 8 most common FAB types found within this collection. The FAB formula of pEC958, F2:A1:B-, is also the most common replicon type, accounting for 20.6% of all 97 *E*. *coli* ST131 strains, or 27.8% of clade C strains, all of which also belong to subclade C2. The second most common type is F1:A2:B20, of which 17 are in subclade C1 and 1 is in clade A. In terms of individual replicons, FIB is present in 100% of clade A and B strains, while FII is most common in clade C (87.5%; S1 Table). Based on our sequence analysis, 3/97 strains do not harbor an IncF plasmid.

**Table 2:** Prevalence of IncF plasmid types in *E*. *coli* ST131 strains.

	*E*. *coli* ST131			Total	
FAB formula^a^	Clade A (n = 9)	Clade B (n = 16)	Clade C (n = 72)	(n = 97)	%
F2:A1:B-			20	20	20.6
F1:A2:B20	1		17	18	18.6
F-:A-:B10	2	7		9	9.3
F22:A1:B20			6	6	6.2
F36:A-:B1			5	5	5.2
F2:A-:B1		4		4	4.1
F48:A1:B26			4	4	4.1
F2:A-:B-			3	3	3.1
Others^b^	5	5	15	25	25.8
Not IncF	1		2	3	3.1

^a^ We used FAB formula to indicate FII, FIA and FIB alleles found in each strain. It does not imply that these alleles are located on the same circular plasmid DNA molecule.

^b^ There are 27 unique FAB types found in 97 strains. Eight most prevalence types are presented here, the remaining are provided in S1 Table.

## Discussion

Our study presents a full annotation of pEC958, a multi-drug resistance plasmid in the well-characterized *E*. *coli* ST131 strain EC958 [18, 19, 23]. In addition, we identified genes required for the maintenance and stability of pEC958. Although IncF plasmids are extremely successful in the *E*. *coli* ST131 clonal lineage [67], this is the first study to examine the biology of an IncF plasmid in its native host using TraDIS [68]. The replication and stability of IncF plasmids (F-plasmid, R1, and R100) has been well documented [39, 47, 69, 70]. Here we provide insights into the interplay between two replicons in order to achieve stable maintenance of the circular plasmid DNA on which they co-exist.

The data analysis in this study used a straight cut-off based on the insertion index of the *sopAB* genes, which encode the partitioning system of pEC958. Mutation of *sopAB* is known to cause destabilization of IncF plasmids and thus they represent characterised genes involved in plasmid stability [71, 72]. This deviation from the model-based approach, in which the cut-off is defined as the intercept of two distribution models representing essential and non-essential genes [23], was chosen because of two reasons: (i) the number of genes on plasmid is insufficient to build two distribution models; and (ii) the cut-off previously defined using chromosomal data is not applicable because of the higher insertion frequency on the plasmid (i.e. one insertion every 4.96 bp compared to every 9.92 bp on the chromosome). In the case of the well-characterised IncF system, use of a straight cut-off assumed that any gene with an insertion index lower than the *sopAB* genes would have a similar or stronger effect on plasmid stability. The stable maintenance of large plasmids such as pEC958 is achieved by the contribution of multiple factors, including systems involved in replication, partitioning and toxin-antitoxin production. Using the strategy outlined, we aimed to identify genes that when mutated caused destabilization of plasmid pEC958—thus they must play a role in plasmid stability.

Our results showed that RepFII, particularly the *copA*, *repA1*, *repA4* genes and *oriV* region, is required for the replication of pEC958. This is consistent with previous studies on the function of RepFII in the IncFII plasmid R100 [41]. In contrast to R100, the RepFII region on pEC958 does not contain its own intrinsic partition system (*stb* locus on R100 [73, 74]). Furthermore, we could not identify any region that resembles a partition site (centromere-like) elsewhere on pEC958 other than within the RepFIA region. Thus, it is reasonable to assume that the *sopAB* genes in the RepFIA region [75, 76] represent the only active partition system on pEC958. Indeed, our transposon mutagenesis revealed a very low insertion index for both *sopA* and *sopB*, confirming the requirement of these two genes for pEC958 partitioning and allowing us to use these genes as a reference threshold to identify biologically significant genes required for plasmid maintenance.

Using TraDIS, we were able to demonstrate that none of the known replication genes in RepFIA are required for pEC958 replication. This included the *oriV-1* of RepFIA, which was not expected to be functional due to the absence of the *repC* gene [40]. The *oriV-2* and its associated genes in RepFIA appear to be intact yet dispensable in pEC958. Similar behavior has been reported in the dual-replicon plasmid pCG86, which contains an active RepFII replicon and an inactive (but intact) RepFIB replicon [77]. This is consistent with a previously proposed model for plasmid speciation, in which the existence of co-integrate plasmids (such as pEC958) allows one replicon to be relaxed and free to accumulate mutations whilst the other replicon is constrained by evolutionary pressure to maintain its replication function [78].

The RepFIA also carries one toxin-antitoxin system *ccdAB* in which the antitoxin CcdA is protected from transposon mutagenesis, indicating that the system is active in pEC958. There are three other TA systems in pEC958, none of which were required for plasmid stability under the conditions tested in this study. Others have suggested that TA systems are more than just plasmid maintenance systems; they can also function as a stress-response system [79, 80], as a programmed cell-death network [81], or as a reversible bacteriostasis system (i.e. induction of dormancy or persistence) [82, 83]. It is conceivable that the redundancy of TA systems on pEC958 is linked to other functions that provide a fitness advantage to its host.

Plasmids of several different incompatibility types have been identified in *E*. *coli* ST131, including IncF, IncI1, IncN, IncA/C and *pir*-type [2]. Our data demonstrates that IncF plasmids are the most common plasmid type in *E*. *coli* ST131, and is in accordance with previous studies [2, 4]. To investigate the prevalence of pEC958 sequences in our strain collection, we used genome sequence data to evaluate the prevalence of pEC958 genes and to perform *in silico* IncF replicon sequence typing. We identified 20 strains (including EC958) that contained more than 70% of the genes identified on pEC958, suggesting that many ST131 strains carry very similar plasmids. We also identified 20 strains that possess the F2:A1:B- plasmid replicon formula, 17 of which contain >70% of pEC958 genes. Taken together, our data demonstrate that pEC958 belongs to the most common group of IncF plasmids found in *E*. *coli* ST131.

The overall success of IncFII plasmids extends beyond the carriage of *bla*_CTX-M-15_ in *E*. *coli* ST131. IncFII plasmids that have acquired the *bla*_NDM-1_ gene (thus conferring carbapenem resistance) have been described in the ST131 lineage [11, 84], but strain EC958 was isolated prior to the discovery of NDM determinants and we did not find any NDM determinants in the 97 ST131 strain collection. The IncFII_k_ plasmid, a replicon type originally found in *Klebsiella* [26], has also been found in KPC-producing ST131 strains in the USA and China [85, 86]. The evolution and continual gain of new antimicrobial resistance determinants in IncFII plasmids represents a major challenge for our understanding of plasmid biology and the spread of antibiotic resistance genes. Here, we shed novel insight into our knowledge of plasmid replication by providing direct evidence that the RepFIA and RepFII replicons in pEC958 cooperate to ensure their stable inheritance. The combination of replication from RepFII and partition from RepFIA may represent a co-evolutionary adaptation for this common plasmid type.

## Supporting Information

S1 Table The presence/absence of pEC958 coding sequences in *E*. *coli* ST131 strains. (XLSX)
